# Editorial: Role of Astrocytes in Seizures Induced by Traumatic Brain Injury

**DOI:** 10.3389/fneur.2020.604788

**Published:** 2020-10-22

**Authors:** Fushun Wang, Fang Pan, Hajime Hirase, Jason H. Huang

**Affiliations:** ^1^Institute of Brain and Psychological Science, Sichuan Normal University, Chengdu, China; ^2^Department of Medical Psychology, Shandong University Medical School, Jinan, China; ^3^Center for Translational Neuromedicine, University of Copenhagen, Copenhagen, Denmark; ^4^Department of Neurosurgery, Baylor Scott & White Health, Temple, TX, United States; ^5^Department of Surgery, Texas A&M University College of Medicine, Temple, TX, United States

**Keywords:** epilepsy, astrocyte, traumatic brain injury, seizures, inhibitory neurons, glymphatic system

Epilepsy is a common chronic neurological disorder affecting more than 1% of the population. Epileptic seizures are uncontrolled sudden attacks of a convulsive or non-convulsive nature, associated with intense neuronal firing. The cellular mechanisms underlying seizure activity are incompletely understood, therefore hindering current medical management. Traumatic brain injury (TBI) is a leading cause for seizures, which offers a good model for studying the mechanism behind epilepsy. TBI patients may exhibit seizures within a week or following several years after injury (1), both cases being related to the severity of TBI. However, mechanisms for acute and chronic seizures may differ, as inflammation-induced factors play an important role in acute seizure, whereas a spatially restricted seizure focus in the brain can often be identified for chronic seizure.

Almost all impairment to the brain results in reactive gliosis (astrocytosis), which is characterized by severe morphological and biochemical changes of pre-existing astrocytes as well as the generation of new astrocytes. These astrocytes form a dense scar tissue that has been suggested to be the epileptic focus. This suggests that glial scar might have an important role in the seizure (2). After injury, the astrocytes demarcate the lesion area and separate the injured tissue from its surroundings. Activated astrocytes produce a diffusion barrier for molecules that are potentially harmful the spared tissue and thereby attenuating the spread of neurotoxicity and preventing excitatory amino acid induced neuron death. The glia scar consists predominately of reactive astrocytes, microglia and extracellular matrix molecules, such as chondroitin sulfate proteoglycans. These cells provide tropic and metabolic support to prevent secondary degeneration. Similar to their role in healthy tissue, astrocytes provide tropic support at the injury site. This can be crucial for the surviving neurons, which locates close the scar.

Metabolites, and growth factors are produced by astrocytes and support the viability of the nearby neurons (4). Historically, glial cells were thought to provide only metabolic and physical support for neurons, serving as the primary source of energy for neurons and serving to control ionic homeostasis and neuronal excitability by buffering K^+^. Current research has expanded our knowledge and found that astrocytes can actively regulate these processes, activated by agonist-induced Ca^2+^ waves (3). Astrocytes are also actively involved in the maintenance of the blood-brain barrier, regulating water and ion homeostasis and amino acid neurotransmitter metabolism, as well as energy and nutrient support of neurons. Many properties of astrocytes also make them important targets for the developing field of epilepsy treatment, and significant advances have been made in epilepsy research in the last decades.

In all, multiple lines of evidence support the contribution of astrocytes for both acute and chronic TBI-induced seizure. Astrocytes are the most numerous glial cell type and account for one third of brain mass. In this Research Topic, we welcomed research studies exploring the role of astrocytes in TBI-induced seizures, and we have got 16 submissions and eight papers are peer-reviewed and accepted.

In the paper titled “Role of Astrocytes in Post-Traumatic Epilepsy,” the authors Xu et al. made a comprehensive review about the complex neurological changes in astrocytes after traumatic brain injury. They described the changes of cell morphology, neurotransmitters, biochemistry, and cytokines in astrocyte during post-traumatic epilepsy. In addition, they also discussed the relationship between dynamic changes in astrocyte and seizures and the current pharmacologic agents used for treatment.

In the paper titled “Identification and management of paroxysmal sympathetic hyperactivity after traumatic brain injury,” the authors Zheng et al. reviewed recent studies about the role of paroxysmal sympathetic hyperactivity in traumatic brain injury, and the review paid a lot of attention to the definition and diagnostic criteria, epidemiology and pathophysiology, symptomatic treatment, and prevention and control of paraoxysmal sympathetic hyperactivity (PSH) in TBI patients, and suggested that treatment for PSH may help the treatment from TBI in three main ways: eliminating predisposing causes, mitigating excessive sympathetic outflow, and supportive therapy.

In the experimental paper titled “Omega-3 polyunsaturated fatty acids alleviate traumatic brain injury by regulating the glymphatic pathway in mice,” the authors Zhang et al. studied the role of omega-3 in the treatment of TBI, and they found that Omega-3 can alleviate neurological impairment in TBI by protecting the glymphatic pathway via suppressing AQP4 expression and partially preventing loss of AQP4 polarity in mice undergoing TBI.

In the experimental paper titled “Amyloid beta oligomers target to extracellular and intracellular neuronal synaptic proteins in Alzheimer's disease,” the authors Ding et al. identified three Aβ binding proteins as α3-Na/K-ATPase, synGap and Shank3 that is possibly involved in the TBI induced cognitive dysfunction. Soluble Aβ oligomers appear capable of attacking neurons via specific extracellular as well as intracellular synaptic proteins. Impact on these proteins hypothetically could lead to synaptic dysfunction and loss and could serve as novel therapeutic targets for AD treatment by antibodies or other agents. This suggested a good way to preventing the cognitive dysfunction after TBI.

In the experimental paper titled “Piperine Attenuates TBI induced Seizures via Inhibiting Cytokine Activated Reactive Astrogliosis,” the authors Song et al. investigated the effects of piperine on early seizures in mice after TBI, and to explored the mechanism of the drug against the development on TBI, and they found that piperine treatment can reduce the degree of cerebral edema, down-regulate the TNF-α, IL-1β, and BDNF, decrease the reactivity of GFAP in the hippocampus, and inhibit TBI induced seizures.

In the experimental paper titled “CRISPR/Cas9 Induced Loss of Keap1 Enhances Anti-oxidation in Rat Ad-MSCs,” the authors Zhu et al. used genetic methods to edit Keap1 gene in mesenchymal stem cells and observed their anti-oxidative ability, because the high level of oxidant micro-environment in lesion region lead more than 99% cells into death during TBI. Their study revealed that loss of Keap1 resulted in anti-oxidative ability in Ad-MSCs, suggesting that our strategy can hopefully increase the viability of mesenchymal stem cells after grafting. This study is also a frontier exploration to the application of CRISPR/Cas9 in Ad-MSCs in TBI patients.

In the clinical paper titled “Clinical characteristics of anti-GABA-B receptor encephalitis,” the authors Zhu et al. reported a kind of brain injury induced encephalitis, which is named Anti-GABA-B (gamma aminobutyric acid-B) receptor encephalitis, and found that the main clinical symptoms were frequent epileptic seizures, cognitive dysfunction and mental behavioral disorders. In addition, they found that anti-GABA-B receptor encephalitis mainly occurred in middle-aged men, and MRI or PET-CT revealed abnormal signals and local metabolism, respectively, in the temporal lobe.

In the paper titled “Brain Proteomic Profiling in Intractable Epilepsy Caused by TSC1 Truncating Mutations: A small sample study,” the authors Liu et al. reported a kind of epileptic disease Tuberous sclerosis complex (TSC), which is characterized by seizures, mental deficiency, and abnormalities of the skin, brain, kidney, heart, and lungs. Their results suggested that TSC is inherited in an autosomal dominant manner and is caused by variations in either the TSC1 or TSC2 gene, and TSC1 truncating mutations may affect the pathway of amino acid metabolism. The study provides a new idea to explore the seizure induced by brain damage.

In all, this special topic has collected some interesting papers about TBI induced seizures and hopefully, this collection will provide some recent advances from molecular mechanisms to better therapeutic strategies for post-traumatic epilepsy.

## Author Contributions

FW wrote this article. FP, HH, and JH revised it. All authors contributed to the article and approved the submitted version.

## Conflict of Interest

The authors declare that the research was conducted in the absence of any commercial or financial relationships that could be construed as a potential conflict of interest.
